# Dendritic and Langerhans cells respond to Aβ peptides differently: implication for AD immunotherapy

**DOI:** 10.18632/oncotarget.6123

**Published:** 2015-10-14

**Authors:** Jiang Cheng, Xiaoyang Lin, David Morgan, Marcia Gordon, Xi Chen, Zhen-Hai Wang, Hai-Ning Li, Lan-Jie He, Shu-Feng Zhou, Chuanhai Cao

**Affiliations:** ^1^ Department of Neurology, General Hospital of Ningxia Medical University, Yinchuan, Ningxia, China; ^2^ Department of Pharmaceutical Sciences, College of Pharmacy, University of South Florida, Tampa, FL, USA; ^3^ USF-Health Byrd Alzheimer's Institute University of South Florida, Tampa, FL, USA; ^4^ Department of Molecular Pharmacology and Physiology University of South Florida, Tampa, FL, USA; ^5^ Department of Colorectal Surgery, General Hospital of Ningxia Medical University, Yinchuan, Ningxia, China; ^6^ Department of Endocrinology, General Hospital of Ningxia Medical University, Yinchuan, Ningxia, China; ^7^ Guizhou Provincial Key Laboratory for Regenerative Medicine, Stem Cell and Tissue Engineering Research Center & Sino-US Joint Laboratory for Medical Sciences, Guiyang Medical University, Guiyang, Guizhou, China

**Keywords:** dendritic cell, Langerhans cell, vaccine, amyloid beta peptide, T-cell epitope

## Abstract

Both wild-type and mutated beta-amyloid (Aβ) peptides can elicit an immune response when delivered subcutaneously. However, only mutated forms of Aβ can sensitize dendritic cells when administered intravenously or intraperitoneally. To understand the role of mutation and delivery routes in creating immune responses, and the function of dendritic cells as therapeutic agents, we used fluorescent-conjugated WT Aβ1-40 (WT40) and artificially mutated Aβ1-40 (22W40) peptides to treat dendritic and Langerhans cells from young and/or old mice at different time points. The cell types were analyzed by flow cytometry and confocal microscopy to identify differences in function and antigen presentation, and Luminex and Western blots for cell activation and associated mechanisms. Our results demonstrated that the artificial mutant, 22W40, enhanced dendritic cell's phagocytosis and antigen presentation better than the WT40. Interestingly, Langerhans cells were more effective at early presentation. The artificial mutant 22W40 increased CD8α^+^ dendritic cells, CD8^+^ T-cells, and IFN-γ production when co-cultured with self-lymphocytes and dendritic cells from aged mice (30-month-old). Here, the 22W40 mutant peptide has been found to be potent enough to activate DCs, and that dendritic cell-based therapy may be a more effective treatment for age-related diseases, such as Alzheimer's disease (AD).

## INTRODUCTION

Immunotherapy has been widely implemented in the treatment of various types of diseases, spanning from foreign pathogens to autoimmune origins. Traditionally, immunotherapies can be grouped as either vaccine or antibody/anti-sera therapy. Vaccination, also known as active immunotherapy, works by injecting an antigen into the subject to induce an antibody response to the injected antigen. This method was originally designed for prophylactic purposes because it requires incubation period, about 2 weeks [1] and even longer for the elderly [2, 3],to generate protection. Therefore, vaccination is generally not used while the person is already affected by the pathogen. However, antibody therapy, otherwise known as passive immunotherapy, aims to deliver antibody or antisera into a subject who may be already affected by the pathogen. Although passive immunotherapy works ideally for those already showing disease symptoms, long term use of this method can induce serum sickness, where the body produces antibodies against the injected sera. In addition, the relatively high cost associated with this method limits its preference.

With advancements in technology, vaccination methods have been expanded to treat many other diseases such as cancer, diabetes, and arthritis. An HPV vaccine that was recently licensed is currently making strides at eliminating HPV-derived cervical cancers [4]. Almost a decade ago, there was a breakthrough in neurodegenerative disease vaccine development when Elan pharmaceutical launched the first clinical trial of Alzheimer's vaccine developed from Aβ peptide plus a strong adjuvant. Although the clinical trial was suspended by the FDA due to strong adverse effects [5, 6], the fundamental work opened a new era for immunotherapies against neurodegenerative diseases. Currently, the idea of immunotherapy has been extended to any therapy that works via modulation of the immune system, and from this, we are starting to see vaccines become less of a prophylactic measure to more of a treatment procedure.

Cell-based immunotherapies have become a dominant therapeutic method for cancer because of its self-donor property. Based on this same property, the approach has been evaluated for AD treatment in mouse model [7, 8]. With progress in knowledge on dendritic cells, more potent therapeutic vaccines have been developed for use in disease treatment. Dendritic cells (DCs) are considered as the professional antigen presenting cells (APC), and play a very critical role in antigen presentation to the immune system. They also serve as mediators between the innate immune system and the adaptive immune system. The role of APCs in neurodegenerative diseases is scarcely studied, and so, understanding the role and properties of DCs in AD will help us better unravel the mechanism for Alzheimer's disease progression, thus hopefully leading to a solution.

Both mature and immature DCs can be found in the circulatory system; they are specialized for antigen uptake, procession and presentation to T-cells [9, 10]. For a long time, immunologists have believed that DCs from peripheral blood were the same as those residing in the skin, known as Langerhans cells (LCs). However, DCs in the blood comprise of both mature and immature phenotypes, whereas Langerhans cells (LCs) are immature cells of the DC system. LCs also take up antigens, but only in the epidermis. In addition, DCs and LCs carry different surface markers, implying that they may have different functions. Antigen-stimulated DCs and LCs migrate to secondary lymphoid organs to stimulate T-cells, and initiate an immune response. A recent discovery of the existence of lymphatic system in the brain is an impetus for DCs based vaccine for brain diseases [11]. As originally discovered by Alois Alzheimer, the brain of an AD patient is overwhelmed with Aβ-42/43 peptide plaque buildup [12]. This extracellular protein inspired the use of the misfolded molecules, contained in the plaques, as the antigen component of a vaccine. It was discovered that Aβ1-42 peptide contained two very strong B cell epitopes [13], and one major T-cell epitope [14, 15]. It is interesting to note that beta-amyloid peptide sequence is highly conserved among mammalian species [16], thus a prompt that it might have important biological function(s), although this is/ are yet to be precisely elucidated [17]. Not with standing, the peptide has been reported to show some protection against oxidative stress in the brain [18, 19], to aid in cholesterol transport [20], and have antimicrobial activity [21, 22]. The Aβ peptide alone has been shown to induce antibody response, without the use of an adjuvant [23]. This indicates that Aβ1-42 is very immunogenic and may play very important role in immune balance or tolerance. Interestingly, known human mutations in the T-cell epitope of Aβ have been linked to different clinical symptoms: patients with the Dutch mutation predominantly show hemorrhaging in the brain [24, 25], while patients with the Flemish mutation demonstrate both AD-like amyloid deposition and hemorrhaging [26]. It has also been suggested that these mutations in the T-cell epitope enhanced the production of Aβ plaques, and therefore may be directly related to the early onset of the disease [27, 28]. In fact, when mutant Aβ peptide was used as vaccine, it showed more immunogenicity than the wild-type form [29].

Currently, many cell-based vaccines use peptide-sensitized DCs with less focus on LCs. However, most vaccines used for disease prevention are delivered via intradermal injection, and work through the activation of LCs. Thus, it is extremely important to understand the differences between how DCs and LCs work, upon antigen stimulation, when used in vaccination. In our previous study, we proved that the WT form of Aβ peptide is immunogenic and even more profound was the immunogenicity of the mutant variants (Flemish and Dutch) [29]. When we used wild-type and mutant peptide-sensitized DCs as vaccines, cells sensitized with only the mutant peptides could induce antibody response without triggering inflammation [14]. Meanwhile, when the same peptides were subcutaneously injected into mice, both the wild-type and mutant forms induced antibody response [30, 31]. This interesting observation prompted in us the idea that the same peptide may act differently in the body, depending on the route of administration, and that verifying this phenomena may help in finding and developing safe and effective vaccines.

In line with this, we sensitized bone marrow-derived DCs (BMDCs) and skin-isolated Langerhans cells (LCs) with wild-type and mutant (mutated T-cell epitope, which ranges from among amino acids 17 to 24, using both known human and artificial mutations) Aβ peptides to investigate the significance of mutation and delivery route in vaccine development.

## RESULTS

### Antigen presentation ability of BMDCs in youngC57/B6 mice shows no difference between florescent labeled wild-type and mutant Aβ1-40 peptide

**Figure 1:**
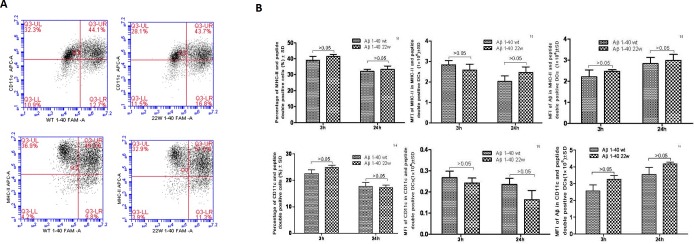
Antigen presentation results of DCs sensitized by wild-type FAM-Aβ 1-40 (WT FAM-Aβ 1-40), and FAM-Aβ40 carrying mutation at aa22 (22W FAM-Aβ 1-40) **A.**, Harvested DCs were identified as MHC class II+ and CD11c^+^ cells using flow cytometry assay after staining with different florescent conjugated antibodies. A (top) is the flow cytometry diagram for antigen stimulated DCs at different time points. Graphs in **B**. demonstrate the percentage of MHCII (top row) or CD11c (bottom row) in the peptide double positive DCs, the mean fluorescent intensity (MFI) of the peptide in the double positive DCs (middle), and the MFI of the MHCII (top right) or the CD11c (bottom right) in the double positive DCs. There is no statistical significant differences between two antigens (*P* > 0.05, *n* = 4).

**Figure 2:**
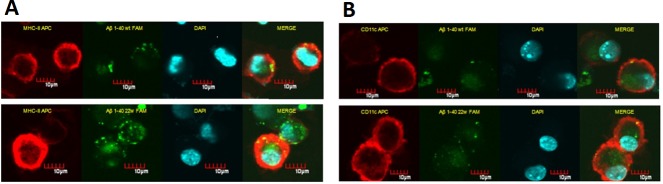
Confocal microscopy images of DCs sensitized by WT and mutant (22W) peptides BMDCs have the ability to uptake and present antigens on the cell surface. The florescent level here is used as indicator for level of antigen presentation. Cells treated the same as in flow cytometry assay, and attached onto slide by cytospin assay: BMDCs stained for MHC-II/CD11c (red fluorescence), incorporated FAM-Aβ40 (green fluorescence). **A.** shows *in vitro* uptake of FAM-Aβ40 WT (top) or 22W (bottom) by cultured BMDCs and the corresponding MHC II levels, where **B.** shows CD11c levels in response to WT (top) or 22W (bottom). In both columns, it seems as if there more localization of MHCII/CD11c with Aβ in mutant peptide-sensitize cells than the wild-type peptide-sensitize cells.

We used bone marrow derived dendritic cells from 2 months old C57/B6 mice, and sensitized them with florescent labeled wild and artificially mutated 22WAβ1-40 peptides. We then analyzed MHC class II and Aβ expression levels at two different time points (12h and 24h). There was no significant difference in the levels of both MHC II and Aβ on DCs treated with either the mutant or wild-type peptide (*P* > 0.05, *n* = 4)(Figure 1A and 1B). To further verify this, we employed confocal microscopy to visualize the location of the antigens. By fluorescence, there seem to be more MHC II/CD11c localization on DCs stimulated with mutant Aβ peptides (Figure 2).

### Langerhans cells (LCs) from young C57/B6 mice show significant differences in antigen presentation ability between florescent labeled wild-type and mutant Aβ1-40 peptide

**Figure 3:**
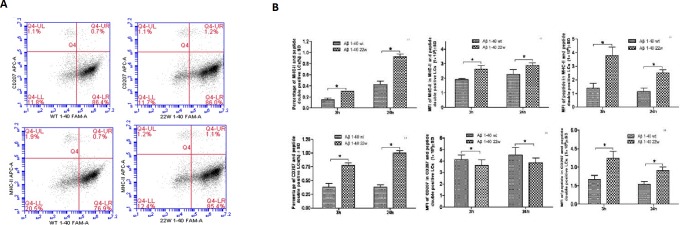
Antigen presentation results of LCs sensitized by wild-type FAM-Aβ 1-40 (WT FAM-Aβ 1-40), and FAM-Aβ40 carrying mutation at aa22 (22W FAM-Aβ 1-40) **A.**, Harvested LCs were identified as MHC class II+ and CD11c^+^ cells using flow cytometry assay after staining with different florescent conjugated antibodies. A is the flow cytometry diagram for antigen stimulated LCs at different time points. Graphs in **B.** demonstrate the percentage of MHCII (top left) or CD207 (bottom left) in the peptide double positive LCs, the mean fluorescent intensity (MFI) of the peptide in the double positive LCs (middle), and the MFI of the MHCII or the CD207 in the double positive LCs. There are significant higher positive cell percentages) and MFI of peptide inside the cells in the mutant peptide treated group than the wild-type peptide treated group (*n* = 4, *P* < 0.05) for both the MHCII and CD207 double positive cells. However, the significances vary for the middle column of graphs comparing the levels of MHCII in the MHCII cells and the levels of CD207 in CD207 cells.

**Figure 4:**
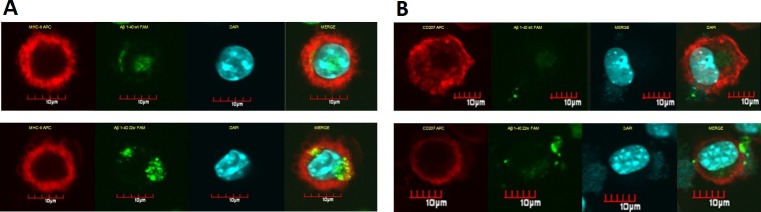
Confocal microscopy pictures of LCs sensitized by different peptides. LCs have the ability of uptake and present antigens on the surface The florescent level here is used as indicator for antigen presentation. Cells treated the same as in flow cytometry assay, and attached onto slide by cytospin assay: LCs stained for MHC-II/CD11c (red fluorescence), incorporated FAM-Aβ40 (green fluorescence). The left column of **A.** demonstrated *in vitro* uptake of FAM-Aβ40 WT (top-left) or 22W (bottom-left) by cultured LCs and studied for MHC-II expression. There seems to be more localization of MHCII with Aβ in mutant peptide-sensitize cells than the wild-type peptide-sensitize cells. The right column of **B.** shows the CD11c expression and Aβ level uptake in the same cell type stimulated with different peptides, either the WT (top) or 22W (bottom) Aβ. There is more CD11c expressed and more antigen in the cell in the mutant peptide-sensitize LCs than in wild-type peptide-sensitize LCs.

When LCs were treated with the same peptide regimen as the DCs, significant differences in the levels of both MHC II and Aβ peptide uptake were observed in a time-dependent manner (Figure 3A, 3B). Additionally, significantly higher double positive cells for CD207 and MHCII were observed (*n* = 4, *P* < 0.05). There were also significant differences in the mean fluorescent intensity (MFI) in the 22W mutant peptide-treated group than their wild-type cohort (*n* = 4, *P* < 0.05). Confocal microscopy confirmed this observation (Figure 4).

### The differences of antigen presentation and T cell activation between DCs and LCs

**Figure 5:**
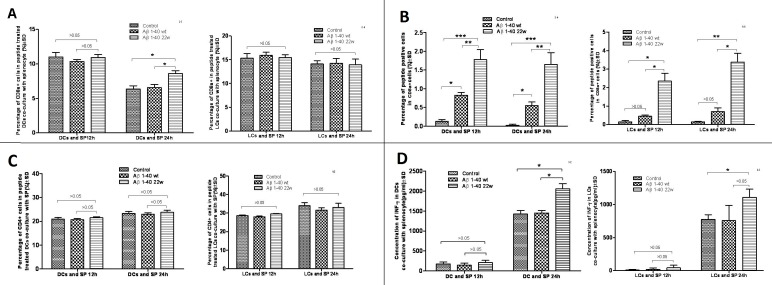
Result of antigen stimulated Dendritic cell (DC) or Langerhans cell (LC) co-cultured with splenocytes When DCs and LCs were prepared from the mouse, the splenocytes were also harvested and frozen. The antigens were presented and allowed for uptake by the cells. Two days after the antigen stimulation to DCs and LCs, the splenocytes were thawed and placed in the incubator overnight. The cells were then co-cultured together on the third day, and cells were harvested next day and an antibody cocktail was used to stain the cells. In **A.**, the percentage of CD8a+ DCs and LCs when co-cultured with splenocytes at 12h and 24h. At 24 hours, there was significant percentage changes in the DCs-splenocytes co-cultured group between two peptides (mutant higher than wild-type and control, *n* = 4, *P* < 0.05) and there is no differences seen in LCs-splenocyte co-cultured group between two peptides at either time point. In **B.**, the percentage of peptide+ cells in CD8a+ cells was measured in the DCs and LCs co-cultured at 12h and 24h. Significances were found for both LCs and DCs cultures between the control, WT, and mutant peptides (*n* = 4, *P* < 0.05), except for when comparing the control and WT groups in LCs (*P* > 0.05). In **C.**, the percentage of CD4+ T-cells were studied in the DCs and LCs co-cultures with splenocytes at 12h and 24h. No significances were found. In **D.**, the concentration of IFN-gamma was studied in the co-culture system at 12 hours and 24 hours. There is a significant difference in the DC population between the control and mutant antigen at the 24h time point (*n* = 4, *P* < 0.05). There is also a significance between the mutant and control antigen in the LC population (*n* = 4, *P* < 0.05).

To identify the ability of antigen presentation, antigen sensitized DCs or LCs were co-cultured with splenocytes. DCs and LCs were allowed 12 and 24 hours to uptake either the control, WT or 22W peptides in a cell culture and then co-cultured with splenocytes. Cell surface marker analysis on these various cells by flow cytometry revealed that the percentage of CD8α^+^ cells was higher in the 22W-stimulated DC-splenocytes co-culture group as compared with the control group after 24 hours of incubation (Figure 5A, left graph). The LC group, however, did not show any significant differences between the groups (Figure 5A, right graph). This indicates increased uptake activity of DC cells when challenged with 22W mutant peptide as compared with either control or WT peptides. The percentage of peptide^+^CD8α^+^(double-positive) cells was significantly higher in the mutated group among all peptide groups at all-time points in the DC culture (Figure 5B, left graph). Though in the LC grouping, significant differences were found between the 22W and the control, and the 22W versus the WT peptides at 12h and 24h, none was found between the control *vs*. the WT peptides at either time point (Figure 5B, right graph). The percentage of CD4^+^ cells showed no significant differences among all the groups and time points for either the DC or LC groupings (Figure 5C). Finally, we measured the concentration of IFN-γ in the co-culture supernatant. There were significantly higher levels in 22W-DC samples than in WT-DC at 24h time point, and higher levels in the 22W-LC than in the control-LC during the same period (Figure 5D).

### Aged mice have the ability to differentiate DC, and mutated peptide better sensitize them

**Figure 6:**
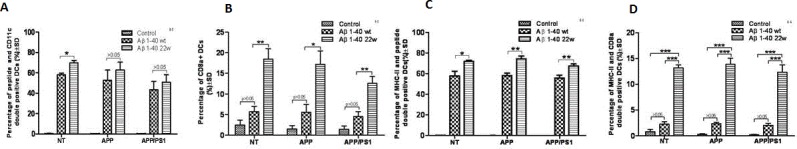
Antigen presentation by DC cells from different genotypes of 30 month old mice The percentage of the CD11c+peptide+ double positive **A**., CD8a+ **B.**, MHCII+peptide+ double positive **C**., and MHCII+CD8a+ double positive **D** cell types were studied after antigen stimulation to DCs cells from non-transgenic (NT) mice, APP, and APP/PS1 mice genotypes (*n* = 4 per group). In studying the CD11c+ cells **A**., we found significance between the WT and mutant (22w) in the NT mouse genotype only (*n* = 4, *P* < 0.05). However, significance was found in all three mouse genotypes, between the levels of WT and 22w Aβ, in the percentages of CD8a+ cells **B**., in the percentages of MHCII+peptide+ cells **C**., and in the percentages of MHCII+CD8a+ cells **D**. (*n* = 4, *P* < 0.05).

After demonstrating the existence of differential antigen presentation by DCs of young mice, we wanted to know the functional activity of these cells in older mice with regards to antigen presentation. Using the same approach as in the young mice, we discovered that older mice (30-months old) still had functional DCs with able antigen presentation ability (Figure 6). Also, the percentages of CD11c^+^peptide^+^ double-positive cells (Figure 6A), CD8α^+^ cells (Figure 6B), MHCII^+^peptide^+^ double-positive cells (Figure 6C), and MHCII^+^CD8α^+^ double-positive cells (Figure 6D) were significantly higher in 22W-mutant DCs than in WT or control peptide treated DCs (*n* = 4, *P* < 0.05).

**Figure 7:**
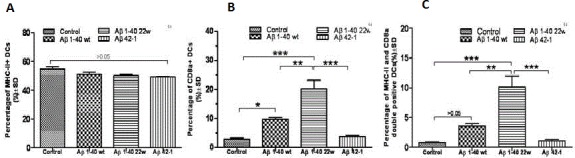
Cell marker induction shows antigen specificity in 30 month old APP/PS1 mice MHCII+ and CD8a+ Dendritic cells (DCs) in the aged mice were studied by flow cytometry (not shown) after antigen stimulation for 24 hours. There was no significant difference when looking at the percentage uptake of MHCII+ DCs in terms of the different peptides **A**., However, significant differences were found in **B.**, when looking at the differences in CD8a+ DCs between the mutant peptide and all over levels (*P* < 0.05). In **C.**, significant differences were also found in CD8a+, MHC-II+ double positive DCs when comparing the mutant peptide to all over levels (*P* < 0.05).

We also included an Aβ 42-1 peptide, a reverse of the normal Aβ 1-42 peptide sequence, as a control antigen to stimulate the DCs from old mice. This was to help clarify whether the observed antigen response was Aβ-specific or just a general antigenicity response due to declined immune function. We discovered that only the Aβ 1-40 WT and Aβ 1-40 22W could successfully sensitize DCs (Figure 7 A, 7B, 7C). There were significant differences in percentages of CD8α^+^ cells (Figure 7B) and MHCII^+^CD8α^+^ double-positive cells (Figure 7C). The 22W mutant peptide significantly activated these aged DCs than any other peptide treatment in this study.

### No differences in inflammatory cytokine secretion by antigen sensitized DCs

**Figure 8:**
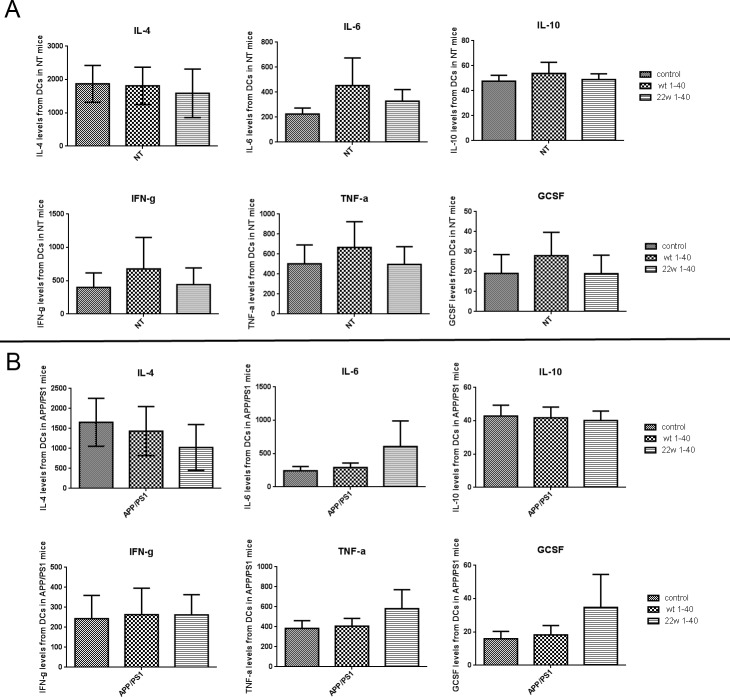
Result of cytokine and chemokine expression profile after antigen stimulation to DCs from different genotype mice Various cytokine levels of dendritic cells (DCs) were detected with Luminex multiplex assay 24 hours after Aβ1-40 WT or Aβ1-40 22w stimulation. The cytokines IL4, IL6, IL10, IFN-gamma, TNF-alpha, and G-CSF levels were measured from DCs cell supernatant of non-transgenic mice (NT) **A.** and APP/PS1 mice **B**. There are no significant differences found among any of the groupings (*n* = 4, *P* > 0.05).

There were significant immune response differences when Aβ1-40WT and Aβ1-40 22W were used to sensitize DCs as a vaccine. To investigate the role of specific cell population in the immune response, we examined cytokine production by DCs from mice of different genotypes (Figure 8). Overall, there was no significant, but just marginal, differences attributed to genotype and peptide stimulation (*n* = 4, *P* > 0.05).

### Peptide sensitized DCs vaccine rely on the mutated T cell epitope but not the MHCI affinity and peptide aggregation

**Figure 9:**
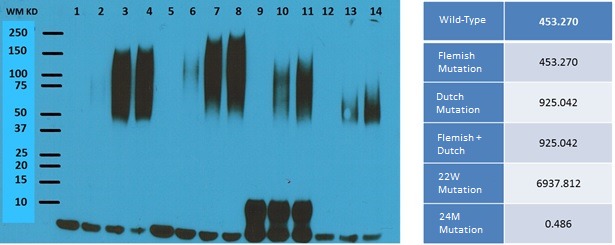
Western Blot results of different types of peptide treatment Shown is the Western Blot result for different mutations in the beta amyloid peptide. The lanes for the western blot result are as follows (all reconstituted from HFIP treated film): Lane 1, WT Aβ without aggregation; Lane 2-4, WT Aβ aggregated for 3 hr, 16 hr, and 24 hr at 37°C; Lane 5, Aβ with Dutch mutation without aggregation; Lane 6-8, Aβ with Dutch mutation aggregated for 3 hr, 16 hr, and 24 hr at 37°C; Lane 9, Aβ with a 22W mutation without aggregation; Lane 10-11, Aβ with a 22W mutation aggregated for 16 hr and 24 hr at 37°C; Lane 12, Aβ with a 24M mutation without aggregation; Lane 13-14, Aβ with a 24M mutation aggregated for 16 hr and 24 hr at 37°C. On the right are the MHC Class I affinity results for each of the different mutations. Each of the rows is as follows: From Top to Bottom, Wild-type, Flemish, Dutch, PFDM, 22W, and 24M.

Western blot analysis was performed on a number of different peptides based on different mutations in beta amyloid (Figure 9). The lowest affinity mutations (24M mutation) showed the highest levels of aggregation (Lanes 12-14), and the WT (Lanes 1-4) had higher pro-aggregation property compared with 22W mutant (Lanes 9-11), as demonstrated by the number of oligomer isoforms from the Western blot. The WT form of Aβ also showed much lower affinity for MHC Class I.

## DISCUSSION

Many studies have reported the link that exists between the immune system and neurological disease, including AD [32-35]. Currently, mounting research have focused on immunotherapies and T-cell therapy as major approaches to treating these disorders [7, 8, 36]. It has been suggested that targeting the immune system may be the safer and more effective treatment approach for AD [37, 38]. With regards to this treatment, dendritic cells (DCs) are the favorable cell types used in immunotherapy, because they can modulate both the innate and acquired immune systems with autologous cells, and because of this, is currently being used exponentially in treatment of disease, such as cancer [39, 40].

About 15 years ago, injection of a novel Aβ peptide vaccine generated great interest in the fields of neuroscience and AD, showing promise in basic research but this was suspended in clinical trial due to adverse effects. Recently, our lab developed a novel and safe vaccine against AD using peptide-sensitized DCs [14, 41-44]. Since then, we have also focused and established a BMDC culture method and repeatedly tested our peptide-sensitized DCs as a therapeutic vaccine for AD. The purity of the DCs derived with this method can be more than 95% when detected with MHCII and CD11c as markers [14, 41, 42]. In the vaccine development process, we also noticed that WT and mutated (22W) Aβ could induce immune response when delivered by subcutaneous injection, with or without an adjuvant. However, the WT peptide failed to induce the same response when used to sensitize DCs alone, and that all peptides with a mutated T cell epitope could induce antibody response, thus the reason for focusing on 22W peptide only in this study. We also investigated difference in activation of DCs and LCs by the wild type and mutant type peptides.

To elucidate the function of our artificial mutant peptide in DCs sensitization and the differences between BMDCs and LCs, we conducted several experiments on both young and old mice to evaluate the function of these cells in order to address this age related disease (AD). There was no significant difference in antigen presentation (Aβ florescence) or MHCII expression level between wild-type and mutant peptide-sensitize DCs (Figure 1A, 1B), but we did observed slightly more fluorescence from the mutant peptide-treated cells (Figure 2). Our hypothesis is that no difference was observed between the WT and 22W mutant peptides because of the relatively young age of the mice. At this age, the immune system is in its prime, and that both peptides are processed with high efficiency. Based on our lab's previous experience, there should be a difference in antigen presentation between the two peptides when mice are treated more frequently and over a longer period of time [31].

When these same peptides were tested in much older mice (30 month old, (Figure 6), the 22W mutant peptide strongly sensitized DCs than WT peptide (Figure 7). Here, we assert that possibly, the 22W mutant peptide elicited such higher response because it could break DCs’ tolerance to Aβ peptides.

Age-related diseases, like AD, show impaired immune function [45, 46], and the immune response in older subjects will most likely be too weak for the body effectively recognize antigens. Therefore when designing a vaccine, a strong adjuvant is usually introduced to help elicit the immune response. This sometimes is too strong in effect, causing massive adverse side-effects as seen in the original Elan pharmaceutical's vaccine for AD. In our study, we have used peptides to sensitize older, 30 month old, mice and our result showed that they effectively sensitize DCs, and more importantly convert to CD8α^+^ cells. The CD8α^+^ cells can then expand and enhance CD8^+^ T-cell population, causing a strong antiviral and bacterial response. CD8^+^ or cytotoxic T-cells plays critical role in fighting infectious disease and cancer, and as older patients are more susceptible to all kinds of infection, this DC vaccine may have unique overlapping benefits.

When LCs from young mice were examined under the same regimen as the DCs, the mutant 22W peptide elicited significantly stronger immune response compared to the WT peptide (*P* < 0.05, Figures 3A, 3B, and 4). This result was noticeably different from that of DCs, as the LCs seemed to respond better to the mutant peptide in young mice. This observation we explain to be tolerance related. LCs reside in the epidermis, papillary dermis, and mucosa and carry out specific homeostatic function [47, 48] and may have little or no prior exposure to a fairly unseen antigen in the epithelia, the Aβ peptide. Therefore, they are less likely to develop tolerance to the peptide, and thus can be readily activated/sensitized by the peptide. On the other hand, DCs which are within the peripheral circulatory system more often encounter peripheral Aβ in the blood and are able to easily/quickly develop tolerance to this peptide

When LCs and DCs were co-cultured with splenocytes, there was increased uptake in the DC population of 22W than the WT (Figure 5). There was also an increase in CD8α^+^ cells from the DC co-culture, but not the LC co-culture, and a corresponding increase in the peptide^+^CD8α^+^ double positive co-culture (significant across all levels of measurement) and also of the LCs (significant only when comparing the 22W to either the WT or control). We also see LCs as being slightly more potent than DCs here in terms of antigen presentation on the bases of percentage positive cells. However, there were no differences in the population of CD4^+^ cells (Figure. 5C). IFN-γ concentrations in the supernatants from the co-cultures, revealed higher levels in the 22W peptide co-cultures of both DCs and LCs at 24 hours. This is very essential in the demonstration that the mutant peptide promotes cellular, rather than humoral, response as evidenced by the increase in CD8^+^ T-cells and IFN-γ production.

One of the major concerns for immunotherapy is the safety of treatment because of the close link between inflammation and AD. The effect of most vaccinations is to prime the immune system, but an overtly strong response can be life-threatening to the patient. To test the safeness of the immune response, we assessed a panel of humoral and cellular induced cytokines and chemokines to validate the DC vaccine. Cytokine and chemokine expression profile analysis showed that there were no significant changes across the panels (Figure 8). This suggests that our DC-based vaccine may not induce excessive inflammation.

The DC activation, in our experiment, is believed to be MHC-I affinity dependent but not specifically peptide conformation related (Figure 9). The lowest affinity mutations (24M mutation) showed the highest levels of aggregation (Lanes 12-14, Figure 9); the WT (Lanes 1-4) demonstrated much pro-aggregation than 22W (Lanes 9-11). The WT form of Aβ was also much lower in terms of MHC Class I affinity and failed to sensitize DCs when tested in both BALB/c and APP/PS1 as compared with 22W peptide. Inferentially, mutant peptides are likely to effectively activate CD8^+^ T-cells. Also the previous notions, that the WT form of Aβ is the largest pro-aggregation form, might not be entirely true, as we have demonstrated that the larger levels of aggregation were exhibited by the mutant peptide (lanes 12 to 14) by Western blot.

Our data from DCs and LCs sensitized with different antigens have demonstrated that (1) BMDCs from older mice can be sensitized with a specific antigen to facilitate normal presentation function; peptide-sensitized DCs can be a very potent and effective therapeutic vaccine in age-related diseases when treated with the proper antigen, (2) mutant Aβ peptide can stimulate CD8α^+^ DCs at a much higher level; this novel function may boost immune activity and help AD patients to withstand progressing AD symptoms. These 22W peptide-treated DCs may be used as a safe and effective vaccine method for AD, (3) LCs have a quicker and stronger response to the antigen than DCs; LCs and DCs may have different function in immune reaction, and (4) the differences in sensitization of DCs and LCs to WT and mutant peptides can be utilized for future vaccine design.

## MATERIALS AND METHODS

### Animals

Adult (8-week-old) male C57/B6 non-transgenic littermate from APP/PS1 breeding colonies were used. All mice were initially genotyped at the time of weaning, and also before sacrifice. Additional confirmation was done using plasma Aβ 40 level. Mice were housed under a 12 hr light-dark cycle, with ad libitum access to rodent chow and water. All described procedures were approved by the IACUC Committee of the University of South Florida. All animals were housed in the vivarium at Byrd Alzheimer's Institute, Tampa, Florida.

### Reagents

Florescent-labeled peptides (FAM-Aβ1-40, FAM-Aβ1-40 with mutation at aa22, and Aβ42-1) were purchased from Biomer Technology (CA, USA); all antibodies for flow cytometry used in were purchased from Biolegend Inc. (CA, USA). The antibodies were: CD3,clone 17A2,CAT 100220;CD4,clone GK 1.5,CAT 100412; CD8a,clone 53-6.7,CAT 100708; CD205,clone NLDC-145,CAT 138208; CD207,clone 4C7,CAT 144203; CD11c,clone N418,CAT 117310; MHC-II, clone M5/114.15.2,CAT 107614; and IFN-γ, clone XMG 1.2,CAT 505810.

### Cells harvesting, dendritic cells differentiation and cell culture

#### Dendritic Cells (DCs) preparation from mouse bone marrow

DCs were harvested and prepared as previously described [31]. In brief, non-transgenic mice littermates (C57/B6) were euthanatized with carbon dioxide (CO_2_). Leg bones were removed and placed in a dish containing 75% ethanol for 1 min and then washed twice with 1×PBS. Bone ends were removed and the marrow cavity was then flushed with 10% RPMI-1640 (RPMI containing 10% FBS) medium. Aspirates were collected in a 50 ml conical tube and then passed through a strainer to separate the cells. The cells were pelleted by centrifugation at 400×g for 10 min at 10°C, and then 3ml of ACK buffer (160 mM NH_4_Cl, 10 mM KHCO_3_ and 0.1 mM Na-EDTA) added for 60s to lyse red blood cells (RBCs). Then cells were resuspended in 30 ml of RPMI-1640 medium. Afterwards, the cells were transferred to a 6 well-plate containing 3 ml 10% RPMI-1640 and reconstituted to 1×10^6^ cells/ml in RPMI 1640. Plate was incubated at 37°C in a tissue culture incubator.

#### Isolation of splenocyte

Freshly acquired spleens were weighed, minced and immediately pushed through a 40μm sieve to obtain a mixed cell suspension. The suspensions were centrifuged (350g, 5min) and supernatant discarded. ACK buffer was added to lyse the red blood cells. 1×PBS was added to stop the lysis, and the cells counted. The splenocyte suspension was centrifuged (350g, 5min) again and suspended in RPMI 1640 (Sigma-Aldrich) with 10% fetal calf serum (Sigma-Aldrich), 10 u/ml IL-2.5μg/ml Con-A and 1% β-mercaptoethanol, and incubated in 5% CO_2_, at 37°C in an incubator.

#### Sensitization of DCs with Aβ peptides and co-culture with splenocytes: DCs differentiation and maturation

a. On day zero, monocytes from bone marrow were collected and cultured under 5% CO_2_in a 37°C incubator.

b. After 24 h, all supernatants were aspirated to remove all non-adherent cells (lymphocytes, progenitors, etc.). Cells were washed twice with 1XPBS gently. Then, fresh 10% RPMI 1640, containing 10ng/ml murine GM-CSF and 10ng/ml IL-4 (BD-Pharmgen, San Jose, CA) and 0.03% β-mercaptoethanol, was added.

c. On the fourth day, 1 ml of culture media was removed and replaced with fresh 10% RPMI 1640 containing 10 ng/ml GM-CSF, 10 ng/ml IL-4. Wild, mutant types of Aβpeptides and Aβ42-1 were added into the designated well at a final concentration of 20 μg/ml.

d. On the sixth day, spleen cells were thawed, and washed 3 times with 1XPBS. Afterwards, it was adjusted to 2×10^6^ cells/ml and cultured in an incubator.

e. On the seventh day, 200 μl of supernatant was carefully obtained from the DC well and stored in the freezer. Peptide-sensitized DCs were collected and washed with 1×PBS twice, then re-suspended with 1× PBS to 1×10^6^ cells/ml. Next, splenocytes with DCs were mixed in a 12 well plate. The proportion was 1:5, thus 0.6×10^6^ DC was mixed with 2.4×10^6^ spleen cells per well.

f. On the eighth day, 200 μl supernatant was again carefully collected from the DC culture, and then stored in the freezer. Then, all cells were harvested and stained with fluorescent labeled antibodies for FCM (flow cytometer) and LSCM (laser scanning confocal microscope).

#### Epidermal explant culture for LCs

We employed the method described by Sparber et al. [49] with slight modification. Briefly, mouse ears were removed at the base with scissors and rinsed briefly in 75% ethanol, then air-dried on sterile gauze for 20 mins. The ears were split into the dorsal and ventral halves (containing the cartilage) with two strong forceps and then the dermal side placed downwards in 0.8% trypsin solution for 25 mins. After 25mins, the tissue was transferred onto a Petri dish containing 10ml pure FBS. The epidermis was peeled off and the dermal piece discarded. Epidermal pieces were then cultured in 3 ml complete medium in 6-well plates for 3 days at 37°C. Afterwards, the epidermal pieces were removed and the emigrated cells were harvested from the culture medium. Cells were centrifuged at 450×g for 5 min at 4°C and then suspended appropriate medium for counting, and usage in subsequent assays.

#### Sensitizing LC with Aβ peptides

Cells were cultured for 24 h in the CO_2_ incubator, and then used for the following procedure:

On the first day, LCs were transferred into 12-well plate (20×10^4^cells per well).

After 72 h, 1 ml of culture media was removed and replaced with fresh 10% RPMI 1640 containing 10ng/ml GM-CSF and 10ng/ml IL-4. Then, wild type and mutant type forms of Aβ peptides, as described earlier, were added separately to a final concentration of 20μg/ml.

On the sixth day, 200μl of supernatant was carefully collected from the LC well and stored. Afterwards, all cells were harvested, and then stained with fluorescent labeled antibodies for FCM and LSCM analyses.

### Cytokines assay (Luminex)

Supernatants from co-cultures were stored at −80°C after collection until used. A panel of 17 cytokines and chemokines, including IFN-γ, IL-2, IL-4, IL-10, IL-12, and TGF-β were measured using mouse multiplex kits from Affymetrix science(CA, USA). Standard and all samples were prepared according to the manufacture's protocol in a 96-well plate. The plate was read on a Bio-PlxMagpix Luminex 200 reader (Bio-Rad, CA, USA), and then the concentration of each analyte calculated based on the generated standard curve.

### Flow cytometry assay

All antibodies for cell labeling were purchased from Biolegend (CA, USA). After 24-hour co-cultured incubation, the cells were harvested and stained in a total volume of 100 μl with 5μl different fluorescent labeled antibodies. After 30 min of incubation at 4%, the cells were fixed in 4% paraformaldehyde in 1×PBS solution. The cells were washed twice in 1×PBS and detected with a BD Accuri C6 Flow Cytometer (CA, USA).

### Confocal laser scanning microscope

All antibodies for cell labeling were purchased from Biolegend (CA, USA). After 24-hour co-culture incubation, the cells were harvested and stained in a total volume of 100 μL with 5μl different fluorescent labeled antibodies. After 30min of incubation at 4%, the cells were fixed in 4% paraformaldehyde in 1×PBS. About 1×10^6^ cells were collected and cytospinned (1000rpm,10min) onto a slide for confocal imaging with Fluoview FV10i confocal microscope systems (Olympus, PA, USA)

### Statistical analysis

Data are expressed as the mean ± SD and analyzed using one-way analysis of variance (ANOVA), followed by Tukey *post hoc* test using Prism 6.0 (GraphPad Software Inc., San Diego, CA). The level of statistical significance was deemed to be *P* < 0.05.
